# Utility of Blood Parameters to Detect Complications during Long-Term Follow-Up in Patients with Diabetic Foot Osteomyelitis

**DOI:** 10.3390/jcm9113768

**Published:** 2020-11-22

**Authors:** Aroa Tardáguila-García, Yolanda García Álvarez, Esther García-Morales, Francisco Javier Álvaro-Afonso, Irene Sanz-Corbalán, José Luis Lázaro-Martínez

**Affiliations:** Diabetic Foot Unit, Clínica Universitaria de Podología, Facultad de Enfermería, Fisioterapia y Podología, Universidad Complutense de Madrid, Instituto de Investigación Sanitaria del Hospital Clínico San Carlos (IdISSC), 28040 Madrid, Spain; aroa_tg@hotmail.com (A.T.-G.); esthergarciamorales@yahoo.es (E.G.-M.); fraalv@hotmail.com (F.J.Á.-A.); irsanz01@ucm.es (I.S.-C.); diabetes@ucm.es (J.L.L.-M.)

**Keywords:** blood parameters, diabetic foot osteomyelitis (DFO), long-term complications

## Abstract

The evidence is still unclear regarding the role of blood parameters in detecting complications in patients who suffer from diabetic foot osteomyelitis (DFO). In this study, the aim was to identify the capacity of different blood parameters in the diagnosis and prediction of the onset of complications. A cross-sectional prospective study was carried out with 116 DFO patients. The following blood parameters were evaluated during 1 year of follow-up: leukocytes, neutrophils, lymphocytes, monocytes, eosinophils, basophils, erythrocyte sedimentation rate (ESR), glycemia, glycosylated hemoglobin, C-reactive protein (CRP), alkaline phosphatase, albumin, and creatinine. Complication events were assessed for each participant during the study period. We investigated the association between blood parameter values and the onset of complication events by conducting a receiver operating characteristic curve analysis. Eighty-five (73.3%) patients developed complications. Regarding blood parameters, higher values of lymphocytes and albumin were predictive factors at the 12-month follow-up once the ulcer had healed. Higher values of ESR had predictive and diagnostic value for the onset of complication events, and higher values of CRP and hyperglycemia were diagnostic factors since they were elevated during the occurrence of an event. In conclusion, after suffering from DFO, the elevation of lymphocytes, ESR, CRP, albumin, and glycemia could be useful in detecting and diagnosing patients who are likely to develop a complication. Serial blood tests are a useful tool for early detection by healthcare professionals to prevent complications.

## 1. Introduction

Diabetic foot osteomyelitis (DFO) is a frequent complication of diabetic foot and is the most common type of diabetic foot infection [1,2]. Furthermore, DFO and its complications are responsible for many sequelae, such as limb loss or death [3,4].

For decades, studies have explored different tools to diagnose DFO and monitor short-term treatment success [1,5]. Blood parameters are one of these tools, and researchers have been trying to find biomarkers in blood that provide information related to DFO [1,3,5,6,7,8,9].

Among these biomarkers, the most relevant one for DFO diagnosis is the erythrocyte sedimentation rate (ESR) [5,6,7,8]. Several studies suggest that blood inflammatory markers could be predictors of the progress of DFO treatment (with either medical or surgical approaches) [10,11]. The first randomized clinical trial [12] to compare both treatments demonstrated that the combination of wound healing and normalization of inflammatory marker levels after 12 weeks suggests that DFO could be considered to have ceased after antibiotic and surgical treatments.

A recent study analyzed the predictive role of inflammatory markers in the time to heal in DFO managed through either medical or surgical treatment [13]. The study suggested that early and adequate treatment of bone infection is essential in DFO patients, because the data about inflammatory markers are similar in both groups of treatment. Thus, the study concluded that there was not enough evidence to define the prognostic role of inflammatory markers in the healing time of ulcers with complications of DFO, regardless of the treatment administered.

Regarding the prognostic value of blood biomarkers, some studies have attempted to prove their role or even their use as cure markers for DFO [11,12]. The evidence regarding the value of these parameters is still limited, and there is a lack of knowledge about their role in detecting complications after healing in patients who suffer from DFO. In addition, clear criteria have not yet been defined about the curing of DFO, which would help to establish development and resolution indicators based on analytical parameters. Furthermore, the establishment of clear cure criteria would facilitate the treatment and follow-up of this disease, thus reducing treatment costs, amputations, and mortality rates. One unexplored area is the utility of serial blood tests, which may help healthcare professionals to detect a complication before its development. Thus, the aim of this study was to identify the capacity of different blood parameters in the diagnosis and prediction of the onset of a complication event.

## 2. Materials and Methods

This prospective cross-sectional study was conducted between November 2014 and November 2018. We included 116 consecutive DFO patients, with a follow-up period of 12 months after healing at a specialized diabetic foot unit.

The inclusion criteria were as follows: patients with diabetes mellitus; age >18 years; ulcers with an area <5 cm^2^; patients who received surgical or antibiotic treatment for the management of DFO and achieved ulcer healing; and patients who agreed to be included in the study after providing written consent.

The exclusion criteria were as follows: patients with DFO suffering from critical limb ischemia (CLI); patients with necrotizing soft tissue infection; patients receiving treatment that modified the biochemical profile (oral or parenteral corticosteroids, immunosuppressive, or cytotoxic agents); patients with acute Charcot foot; women who were pregnant or lactating; and patients who did not understand the purpose of the study or refused to be included.

A total of five visits were scheduled during the study. The first one was a baseline visit, and the rest were follow-up visits. The visits were distributed as follows:

- Baseline visit;
- Visit 1: after healing;
- Visit 2: 1-month follow-up;
- Visit 3: 6-month follow-up;
- Visit 4: 12-month follow-up.

During the baseline visits, we collected patients’ demographic information and medical history, confirmed the DFO diagnosis, and performed a neurological and vascular examination. Clinical suspicion of DFO was established by a combination of a probe-to-bone (PTB) test and plain radiography [14]. The PTB test was performed using metal forceps (Halsted mosquito forceps), and the result was considered positive when the researcher could feel a hard or gritty surface. We considered plain radiographs (two standard views) to be positive for osteomyelitis if they showed cortical disruption, periosteal elevation, a sequestrum or involucrum, or gross bone destruction. The diagnosis of all patients included in the study was confirmed by positive results of bone culture or histology [5].

The neurological examination was conducted using Semmes–Weinstein 5.07/10 g monofilament (Novalab Ibérica, Alcalá de Henares, Madrid, Spain) for pressure perception and a biotensiometer (Me.Te.Da. S.r.l., San Benedetto del Tronto, Italy) for vibration perception. Neuropathy was diagnosed in patients who felt nothing during one of the two tests [15]. Peripheral arterial disease (PAD) was diagnosed if the patient met the following criteria: absence of both distal pulses (dorsalis pedis and posterior tibial pulse); ankle-brachial index (ABI) < 0.9; and in patients with ABI > 1.4 (non-compressible measurement resulting in medial arterial calcification), we considered PAD with a toe–brachial index < 0.7 and a transcutaneous oxygen pressure (TcPO_2_) < 30 mmHg (using a TCM4 transcutaneous monitor; Radiometer Medical, Brønshøj, Denmark) [16,17]. CLI was diagnosed if the patient met the following criteria: absence of both distal pulses and ankle pressure lower than 70 mmHg or ABI < 0.5 or a toe systolic pressure lower than 50 mmHg [17,18].

Ulcer healing was defined as complete epithelialization of the ulcer, with the skin remaining intact after 2 weeks [19]. After healing, patients were evaluated for 12 months, within four follow-up visits for the purposes of measuring blood parameters by blood tests and recording the presence of any complications associated with the previous process. To determine the levels of blood parameters, we drew blood at visits 1, 2, 3, and 4. The blood parameters analyzed during follow-up visits were leukocytes, neutrophils, lymphocytes, monocytes, eosinophils, basophils, the erythrocyte sedimentation rate (ESR), glycemia, glycosylated hemoglobin (HbA1c), C-reactive protein (CRP), alkaline phosphatase, albumin, and creatinine. Elevated blood parameters were defined as follows: leukocytes > 11 × 10^9^/L; neutrophils > 6.8 × 10^9^/L; lymphocytes > 3.7 × 10^9^/L; monocytes > 1.1 × 10^9^/L; eosinophils > 0.5 × 10^9^/L; basophils > 0.1 × 10^9^/L; erythrocyte sedimentation rate > 20 mm/h; glycemia > 5.6 mmol/L; glycosylated hemoglobin > 7.7 mmol/L; C-reactive protein > 476.2 nmol/L; alkaline phosphatase > 129 UI/L; albumin > 48 g/L; and creatinine > 1.1 mg/dL.

We recorded the following outcomes as complication events: DFO recurrence, new case of DFO, soft tissue infection, ulcer recurrence, re-ulceration, minor or major amputation, death, and other events related to diabetic foot disease. The patients were followed until the end of the follow-up time (12 months), any adverse event that caused premature termination in the study, or death. During the follow-up period, all the patients received the same local management. Customized insoles and therapeutic footwear were used. The patients were then monitored once per month according to the recommendations of international guidelines [20]. Patients were asked monthly about their compliance.

Ethical approval was obtained, and the study was completed in accordance with the ethical standards of the responsible committee (14/485-E). Informed consent was obtained from each patient. The authors declare that they have complied with the code of ethics of the Declaration of Helsinki [21].

Data were entered and processed using SPSS^®^ version 20.0 for iOS (SPSS, Inc. Chicago, IL, USA). Descriptive analyses were performed. For quantitative variables, we calculated the means and standard deviations. For qualitative variables, we calculated the frequency distributions and percentages. A receiver operating characteristics (ROC) curve analysis was performed to compare the development of complication events and blood parameter values. The ROC curve analysis established the area under the curve (AUC). Differences were considered significant at *p* < 0.05 for a confidence interval of 95%. The sample size was calculated using GRANMO^®^ version 7.0 for iOS. As a result, the necessary sample was estimated as 80 patients for a study power of 80.0%, type 1 error of 5.0%, and predictable anticipated loss to follow-up of 15.0%.

## 3. Results

**Figure 1:**
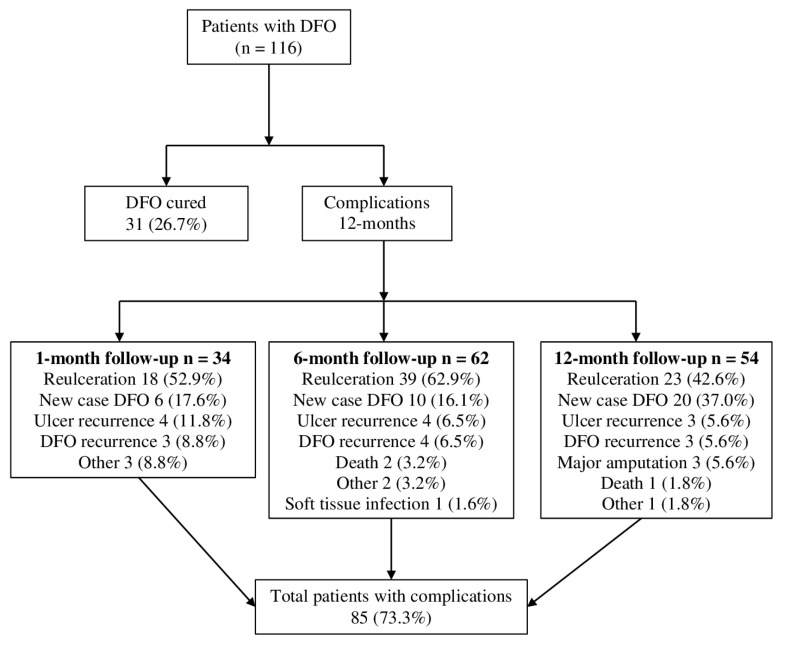
Flow chart of distribution of complications. Abbreviation: DFO, diabetic foot osteomyelitis.

The most frequent complications were re-ulceration and new cases of DFO, followed by ulcer recurrence and DFO recurrence, as shown in Figure 1. During the 12 months of follow-up, a total of 85 (73.3%) patients suffered from some type of complication. Figure 1 shows the distribution of complication events registered among visits. We included 116 DFO patients in the study, and their main baseline characteristics are summarized in Table 1. The blood test data from the follow-up visits are shown in Table 2 and Table 3, which summarize the percentage increase of blood parameters.

**Figure 2:**
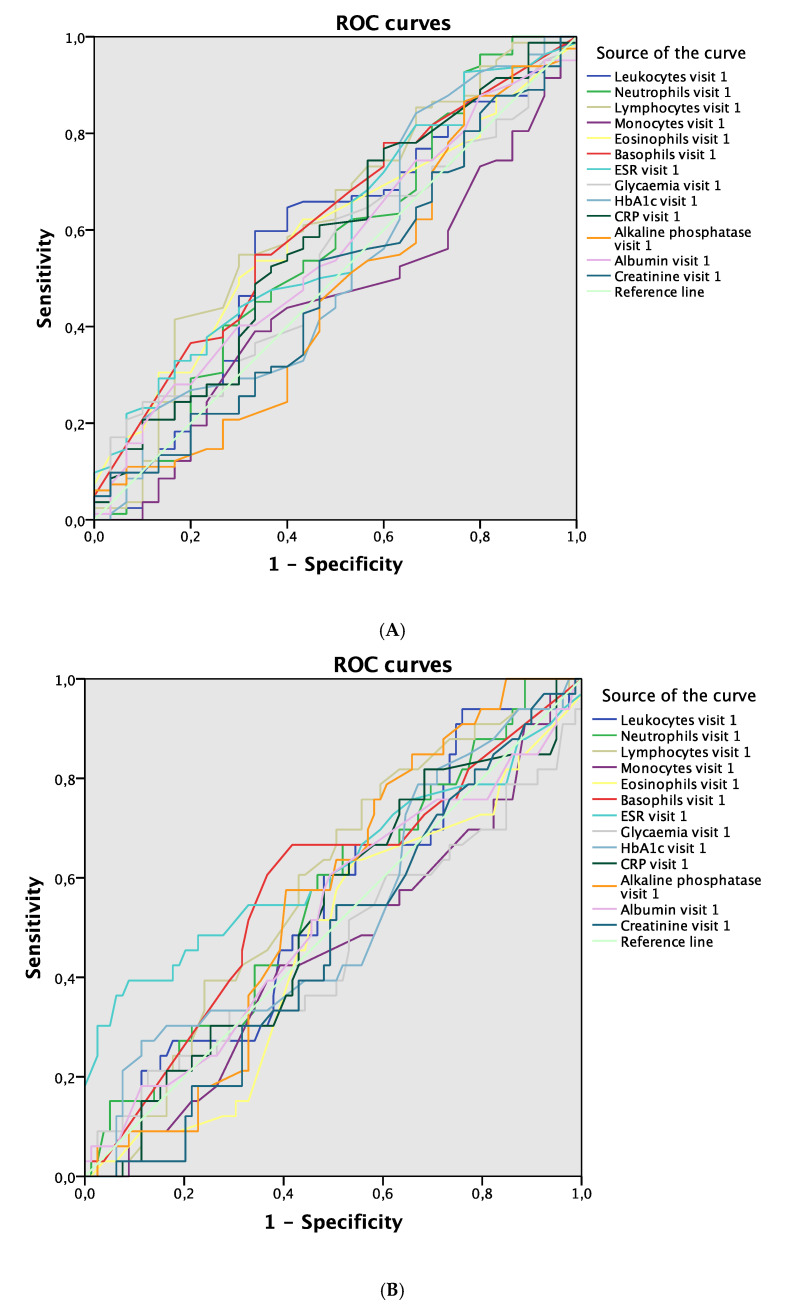

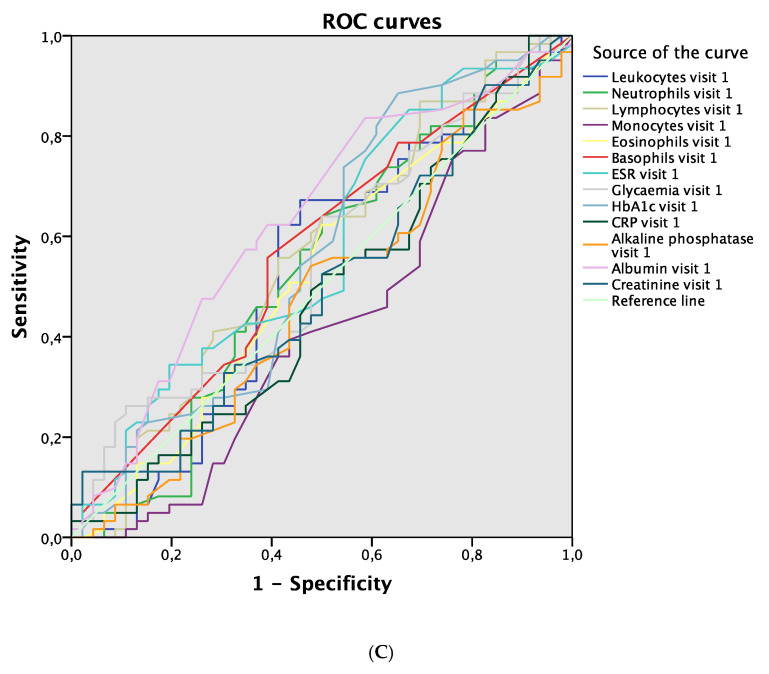
ROC curves. (**A**) Blood parameters in visit 1 associated with complications during 12-month follow-up. (**B**) Blood parameters in visit 1 associated with complications in visit 2. (**C**) Blood parameters in visit 1 associated with complications in visit 3. Abbreviations: ROC, receiver operating curve; ESR, erythrocyte sedimentation rate; HbA1c, glycosylated hemoglobin; CRP, C-reactive protein.

**Figure 3:**
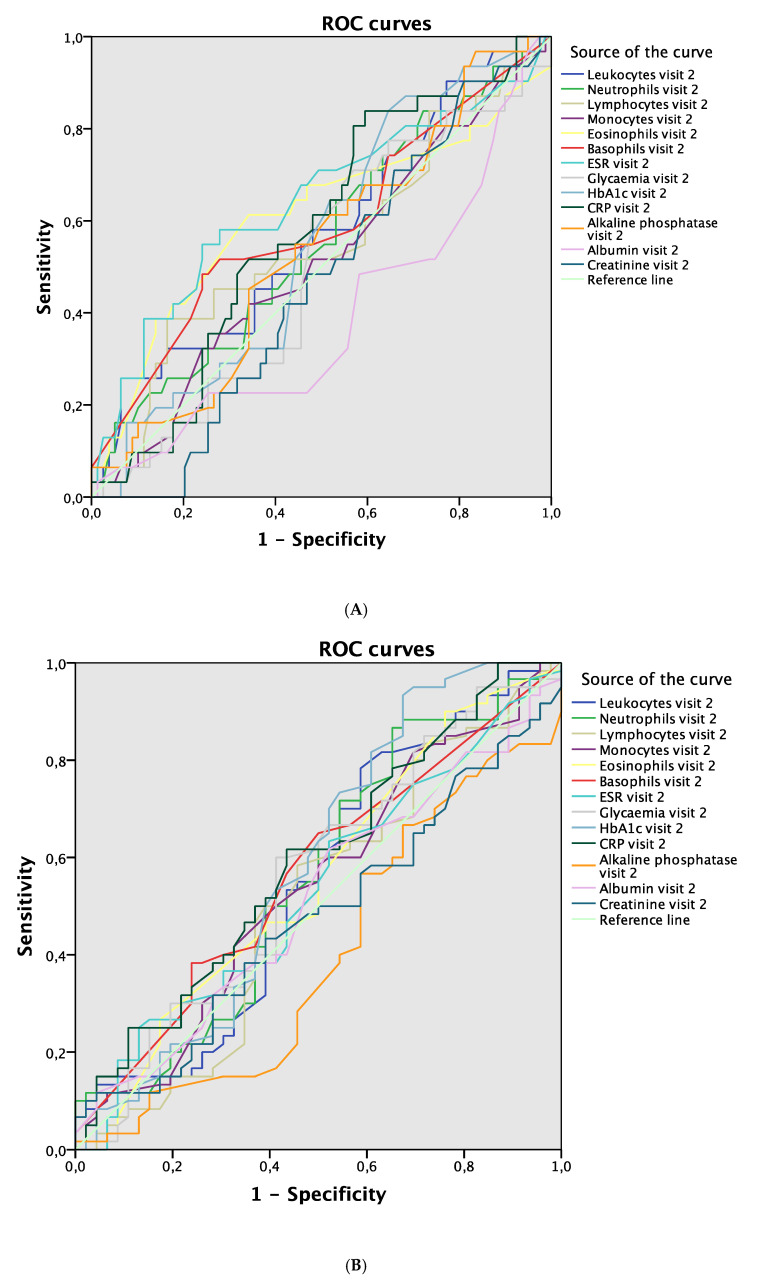

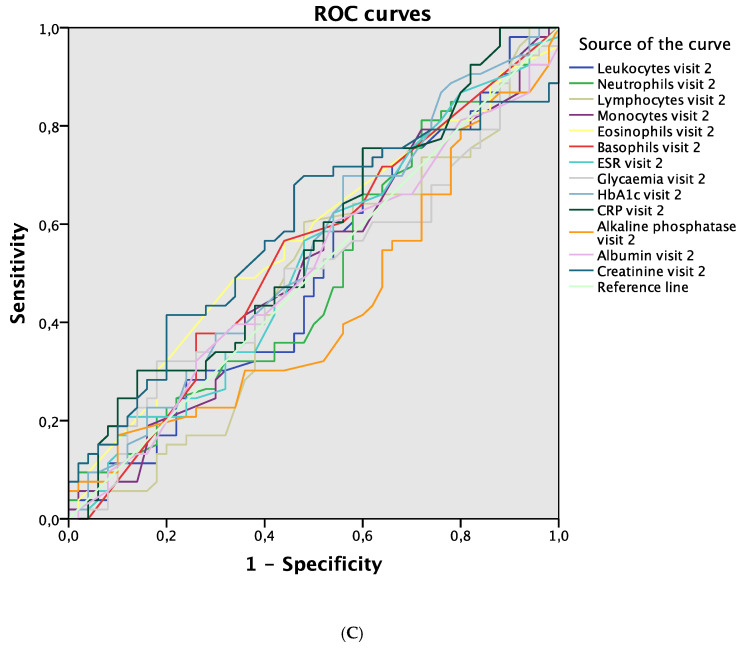
ROC curves. (**A**) Blood parameters in visit 2 associated with complications in visit 2. (**B**) Blood parameters in visit 2 associated with complications in visit 3. (**C**) Blood parameters in visit 2 associated with complications in visit 4. Abbreviations: ROC, receiver operating curve; ESR, erythrocyte sedimentation rate; HbA1c, glycosylated hemoglobin; CRP, C-reactive protein.

Regarding the development of complications according to blood parameter values, lymphocytes were the blood parameter at visit 1 that showed the greatest discriminative power to detect complications during 12 months of follow-up (AUC 0.623; 95% CI 0.502–0.745; *p* = 0.046; Figure 2A). ESR showed the greatest discriminative power in visit 1 for detecting complications after 1 month (AUC 0.623; 95% CI 0.494–0.751; *p* = 0.041; Figure 2B), while albumin showed the greatest power after 6 months (AUC 0.631; 95% CI 0.523–0.739; *p* = 0.020; Figure 2C). In relation to blood parameters evaluated in visit 2, ESR was the blood parameter with the greatest discriminative power for detecting complications in the same visit (AUC 0.640; 95% CI 0.516–0.765; *p* = 0.022; Figure 3A). No differences were found between the values of blood parameters in visit 2 used to detect complications in visit 3 or visit 4 (Figure 3B,C).

**Figure 4:**
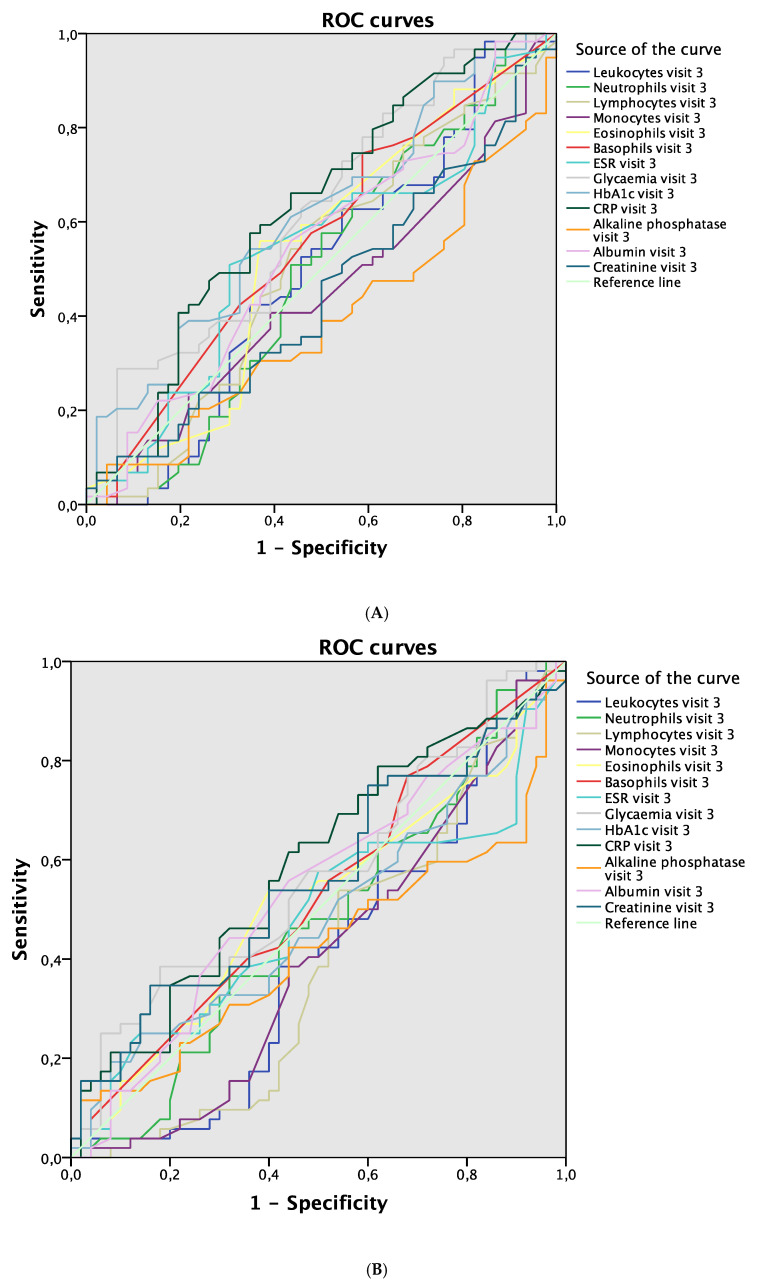
ROC curves. (**A**) Blood parameters in visit 3 associated with complications in visit 3. (**B**) Blood parameters in visit 3 associated with complications in visit 4. Abbreviations: ROC, receiver operating curve; ESR, erythrocyte sedimentation rate; HbA1c, glycosylated hemoglobin; CRP, C-reactive protein.

**Figure 5:**
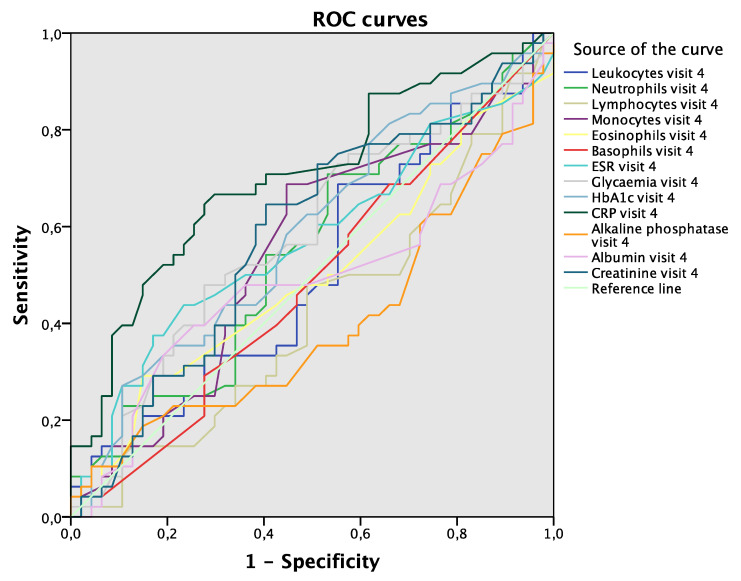
ROC curves. Blood parameters in visit 4 associated with complications in visit 4. Abbreviations: ROC, receiver operating curve; ESR, erythrocyte sedimentation rate; HbA1c, glycosylated hemoglobin; CRP, C-reactive protein.

Hyperglycemia and elevated levels of CRP were the blood parameters in visit 3 that demonstrated the greatest discriminative power to detect complications in the same visit (AUC 0.618; 95% CI 0.509–0.727; *p* = 0.038 and AUC 0.629; 95% CI 0.519–0.738; *p* = 0.024, respectively; Figure 4A). No differences were found between the values of blood parameters in visit 3 used to detect complications in visit 4 (Figure 4B). Finally, elevated CRP in visit 4 was the blood parameter with the greatest discriminative power to detect complications in the same visit (AUC 0.704; 95% CI 0.599–0.809; *p* = 0.001; Figure 5).

## 4. Discussion

Complications after ulcer healing were highly frequent in patients who suffered from DFO, including re-ulceration and new cases of DFO. Our results revealed blood parameters that could be considered predictive of complications and alert us to the development of any complications. We also identified others that can be considered diagnostic factors or confirm the diagnosis of a complication.

Concerning parameters associated with a complication event, we observed that elevated values of lymphocytes and elevated values of albumin were predictive at the 12-month follow-up once the ulcer had healed. The elevation of ESR had predictive and diagnostic value for the onset of a complication event. By contrast, the elevation of CRP and hyperglycemia were diagnostic factors since their elevation occurred during the occurrence of the event.

Blood parameters have mainly been investigated in order to support the diagnosis or monitoring of DFO [1,5,6,7,8,10,11,13,22,23]. However, we have proposed a study that evaluates blood parameters as predictive or diagnostic factors in the development of complications in long-term follow-up after healing in DFO patients. A meta-analysis analyzed blood test data for DFO diagnosis but did not indicate how to use blood parameters for monitoring for DFO cure [8]. Furthermore, the meta-analysis stated that there are insufficient data to support the use of other inflammatory markers, although ESR had the most evidence for DFO diagnosis.

International guidelines [24] suggest that a decrease or normalization of blood parameters in DFO patients could help to stop antibiotic treatment, but there are no specific criteria for stopping the treatment. A prospective study analyzed the effectiveness of blood parameters for the diagnosis and monitoring of DFO treatment and concluded that there is inadequate evidence to support any blood parameter for monitoring DFO treatment [10]. The study concluded that the data are promising but require long-term studies to better define the role of blood parameters in DFO.

A retrospective study found that stagnant values of CRP and ESR are related to poor clinical outcome. Furthermore, both blood parameters suggest a predictive role when monitoring the success of therapy in DFO [11]. However, one important limitation of that study is that it is a retrospective study, because the blood parameter measurements could not be predefined and were different for each patient.

Our data on ESR showed utility for predicting and confirming complications in different follow-up visits. Thus, its evaluation should be standardized during the remission period of DFO, not only for diagnosis or monitoring treatment, as reported previously [8,10,11,23,25]. Furthermore, CRP is a biomarker that is used extensively to support DFO diagnosis [8,23,25], but our findings demonstrated that it is also useful for the confirmation of complications.

The main limitation of our study is that we only tested DFO patients. Moreover, there could be other concomitant risk factors that could influence the development of complications. In future research, it could be interesting to add a control group and analyze the multiple risk factors associated with the development of complications. The main strength of the study is that it had an intensive follow-up program. We consider that this study provides evidence to employ long-term follow-up models for patients who suffer from DFO after healing by performing serial blood tests. The blood parameter data could be useful for the early diagnosis of different complications that could arise during the remission period of DFO.

Serial blood tests are a diagnostic tool that is widely available, easy to perform, relatively cheap, and associated with minimal harm. Thus, they can be used by healthcare professionals to prevent complications. Our results provide sufficient evidence to employ preventive models for monitoring DFO resolution in healing patients. The use of blood tests in selecting patients could be useful in the early detection and diagnosis of complications.

In conclusion, the elevation of lymphocytes, ESR, CRP, albumin, and glycemia could be useful for detecting and diagnosing patients who are likely to develop a complication. Serial blood tests are an easy and useful tool for healthcare professionals to prevent complications by detecting them early.

## Figures and Tables

**Table 1:** Baseline characteristics of the patients.

**Variable**	***n* (%)**		
Gender	Male: 96 (82.8)		Female: 20 (17.2)
Type of DM	Type 1: 12 (10.3)		Type 2: 104 (89.7)
PAD	48 (41.4)		
Neuropathy	116 (100.0)		
Location of the ulcer	Forefoot: 107 (92.2)	Midfoot: 5 (4.3)	Hindfoot: 4 (3.4)
Treatment for DFO	Surgical: 96 (82.8)		Medical: 20 (17.2)
Deformities	90 (77.6)		
Minor amputation	14 (12.1)		
Bone resection/removal	82 (70.7)		
**Variable**	**Mean ± SD**		
Age (years)	62.9 ± 10.1		
DM duration (years)	17.5 ± 12.3		
HbA1c (mmol/L)	10.3 ± 6.7		
Body mass index (Kg/cm^2^)	28.3 ± 5.5		
Duration from ulcer (weeks)	15.7 ± 32.1		
Time until healing (weeks)	15.8 ± 9.7		

Abbreviations: DM, diabetes mellitus; PAD, peripheral arterial disease; DFO, diabetic foot osteomyelitis; SD, standard deviation; HbA1c, glycosylated hemoglobin.

**Table 2:** Blood test—values of blood parameters.

Blood Parameter	Visit 1 Mean ± SD	Visit 2 Mean ± SD	Visit 3 Mean ± SD	Visit 4 Mean ± SD
Leukocytes (×10^9^/L)	8.2 ± 2.4	8.5 ± 2.5	8.3 ± 2.3	8.4 ± 2.3
Neutrophils (×10^9^/L)	4.9 ± 2.1	5.3 ± 2.1	4.9 ± 1.7	5.1 ± 1.6
Lymphocytes (×10^9^/L)	2.8 ± 5.6	2.2 ± 0.9	2.3 ± 1.0	2.2 ± 1.0
Monocytes (×10^9^/L)	0.8 ± 1.3	0.7 ± 0.9	0.7 ± 0.5	0.9 ± 1.3
Eosinophils (×10^9^/L)	0.5 ± 2.5	0.3 ± 0.2	0.3 ± 0.2	0.3 ± 0.2
Basophils (×10^9^/L)	0.1 ± 0.2	0.05 ± 0.04	0.06 ± 0.05	0.05 ± 0.05
ESR (mm/h)	21.7 ± 19.4	21.5 ± 18.6	18.9 ± 21.3	20.1 ± 18.8
Glycemia (mmol/L)	7.9 ± 3.4	8.2 ± 4.1	8.5 ± 3.3	8.0 ± 3.8
HbA1c (mmol/L)	10.0 ± 7.7	10.3 ± 9.5	9.2 ± 0.5	9.2 ± 0.5
CRP (nmol/L)	580.9 ± 952.4	819.0 ± 1971.4	828.6 ± 1733.4	771.4 ± 2000
Alkaline phosphatase (UI/L)	93.7 ± 31.4	90.2 ± 26.8	90.4 ± 26.5	84.0 ± 23.1
Albumin (g/L)	41 ± 5	41 ± 5	41 ± 4	49 ± 82
Creatinine (mg/dl)	1.6 ± 1.6	1.6 ± 1.5	1.7 ± 1.7	1.4 ± 1.1

Abbreviations: SD, standard deviation; ESR, erythrocyte sedimentation rate; HbA1c, glycosylated hemoglobin; CRP, C-reactive protein.

**Table 3:** Blood test—elevated blood parameters.

Elevated Blood Parameter	Visit 1 *n* (%)	Visit 2 *n* (%)	Visit 3 *n* (%)	Visit 4 *n* (%)
Leukocytes	8 (6.9)	18 (15.5)	12 (10.3)	12 (10.3)
Neutrophils	9 (7.8)	13 (11.2)	7 (6.0)	7 (6.0)
Lymphocytes	6 (5.2)	5 (4.3)	3 (2.6)	3 (2.6)
Monocytes	6 (5.2)	8 (6.9)	8 (6.9)	7 (6.0)
Eosinophils	9 (7.8)	5 (4.3)	4 (3.4)	4 (3.4)
Basophils	None	None	None	None
ESR	38 (32.8)	40 (34.5)	29 (25.0)	33 (28.4)
Glycemia	75 (64.7)	73 (62.9)	83 (71.6)	72 (62.1)
HbA1c	96 (82.8)	94 (81.0)	90 (77.6)	86 (74.1)
CRP	33 (28.4)	39 (33.6)	36 (31.0)	28 (24.1)
Alkaline phosphatase	8 (6.9)	7 (6.0)	11 (9.5)	5 (4.3)
Albumin	3 (2.6)	2 (1.7)	5 (4.3)	2 (1.7)
Creatinine	1 (0.9)	42 (36.2)	53 (30.2)	33 (28.4)

Abbreviations: ESR, erythrocyte sedimentation rate; HbA1c, glycosylated hemoglobin; CRP, C-reactive protein.
